# Rapid Relief of Gastroesophageal Reflux Disease (GERD) Symptoms With Sodium Alginate Antacid Suspension: An Indian Real-World Evidence Study

**DOI:** 10.7759/cureus.79991

**Published:** 2025-03-03

**Authors:** Thoguluva S Chandrasekhara, Umesh Chandra Patra, Pradeep Kumar Agarwal, Lalit Shimpi, Kalyan Bose, Sandeep Kulkarni, Dinesh R Patil, Onkar C Swami

**Affiliations:** 1 Gastroenterology, MedIndia Hospitals, Chennai, IND; 2 Hepatology, Srirama Chandra Bhanja (SCB) Medical College, Cuttack, IND; 3 Gastroenterology, Astha Hospital, Dehradun, IND; 4 Gastroenterology, The Cedar Gastroenterology Clinic, Pune, IND; 5 Gastroenterology, Woodlands Multispeciality Hospital Ltd., Kolkata, IND; 6 Gastroenterology, Shree Gastroenterology Clinic, Pune, IND; 7 Clinical Pharmacology, Alembic Pharmaceuticals Ltd., Mumbai, IND

**Keywords:** alginates, gastroesophageal reflux disease, heartburn, real-world setting, regurgitation, sodium alginate antacid

## Abstract

Background and objectives

Gastroesophageal reflux disease (GERD) is a common gastrointestinal disorder characterized by troublesome symptoms affecting the quality of life. Sodium alginate antacid suspension provides quick and prolonged relief of symptoms due to its unique mechanism of action. The primary objective of this study was to evaluate improvement in GERD symptoms by assessing changes in symptom scores at the end of one week and to evaluate the responder rate. The secondary objective was to evaluate patient tolerability using the frequency and severity of adverse events and physician-reported outcomes.

Methods

This was a retrospective, observational data collection study that reviewed medical records of GERD patients who received sodium alginate antacid suspension (10-20 ml, 3-4 times daily) for one week. Data were retrieved, analyzed, translated into symptom scores (GERD Health-Related Quality of Life score), and classified based on disease severity. Primary outcomes were improvement in symptom scores (heartburn, regurgitation, swallowing difficulties, and bloating) and total scores at the end of treatment. The responder rates, physician-reported outcomes, and tolerability were also analyzed.

Results

Medical records for 10,000 patients treated with sodium alginate antacid suspension were reviewed. Out of these, data for 6,246 patients was further analyzed. Treatment with sodium alginate antacid suspension resulted in significant reductions in heartburn, regurgitation, swallowing difficulties, and bloating scores (p<0.001) in patients with moderate to severe GERD. The mean total GERD symptom score also decreased significantly from baseline (p<0.001). The responder rate, i.e., a reduction of ≥50% in the total symptom score from baseline to the end of one week, was 74%. Almost 90% of patients reported symptom improvement, but 2.22% of patients experienced adverse events. As per physicians, this suspension was effective in 96.64% of patients.

Conclusion

This real-world evidence study highlights rapid symptomatic relief in GERD patients with sodium alginate antacid suspension.

## Introduction

Gastroesophageal reflux disease (GERD) is a common gastrointestinal disorder affecting 20% of the population worldwide [1,2]. In India, GERD prevalence varies between 7.6% and 30% [3-5]. It is characterized by the regurgitation of stomach acid contents into the esophagus and presents with symptoms such as heartburn, regurgitation, and chest pain [1,2,6].

GERD represents a significant health concern due to its association with considerable morbidity and diminished quality of life [1,6,7]. Additionally, the symptoms of GERD often lead to sleep disturbances, which can further impact overall well-being [5,7,8]. Effective and rapid symptomatic relief is crucial for managing GERD [1,9]. Alleviating symptoms such as heartburn and regurgitation contribute to a reduction in pain and physical discomfort [1,8]. This, in turn, can lead to better sleep, enhancement in daily functioning, and overall improvement in quality of life [1,9].

GERD is typically managed through a combination of strategies, including lifestyle modifications, dietary adjustments, and the use of potent acid-suppressing agents such as antacids, histamine (H2) receptor antagonists, and proton pump inhibitors (PPIs) [8,10]. Antacids, in particular, offer a means to achieve rapid relief from GERD symptoms by neutralizing stomach acid and providing prompt alleviation of discomfort [10]. Raft-forming antacids represent an ideal choice for managing GERD, considering their unique mechanism of action [11].

Alginate is a key component in many raft-forming systems, a natural polysaccharide polymer extracted from brown seaweed (Phaeophyceae) [11]. When exposed to gastric acid, alginates rapidly form a gel-like raft that targets and displaces the acid pocket distal to the oesophagogastric junction [12-15]. This raft-like barrier protects the esophageal mucosa by preventing acid from refluxing into the esophagus, thereby alleviating GERD symptoms [13,14].

Studies have shown that alginates are particularly effective at managing the acid pocket, a layer of excess acid that forms above an ingested meal [14]. Alginates, when combined with antacids, offer greater relief from heartburn and reduce acid exposure time more effectively than antacids alone [14]. Furthermore, they can enhance symptom control beyond that achieved with PPIs [13,14].

Although numerous studies on using alginate antacids in patients experiencing GERD symptoms yielded promising findings [15-18], there is still a gap in the data that demonstrates its effect on symptom relief in GERD in real-world settings, particularly in Indian patients. Thus, this real-world observational study evaluated the improvement in GERD symptoms in Indian patients receiving sodium alginate antacid suspension for a short duration. The primary objective of this study was to evaluate improvement in GERD symptoms by assessing changes in symptom scores at the end of one week and to evaluate the responder rate. The secondary objective was to evaluate patient tolerability using the frequency and severity of adverse events and physician-reported outcomes.

## Materials and methods

Study design, patient characteristics, and sample size

This was a retrospective, observational, real-world medical record-based study. Medical records of adult patients (age ≥18 years) who consented to the future use of their medical records were retrieved. Data of GERD patients who were previously treated with sodium alginate antacid suspension, were analyzed. The sample size for this data retrieval study was 10,000.

Outcome measurement

Collection of Data

The anonymized records collected for the patients were reviewed. Details of the general examination and GERD symptoms were identified from the medical records. The general examination included measurements of pulse, blood pressure, body weight, and height. GERD symptoms included heartburn, regurgitation, difficulty swallowing, and bloating. Since this was a retrospective study, data for all the parameters for all patients were not available. We have included the data of patients who received sodium alginate antacid suspension, and symptom scores were available.

Translation of Data

The GERD symptoms reported in medical records were translated into symptom scores using the GERD Health-Related Quality of Life (GERD-HRQL) questionnaire [19] parameters. Scores were assigned for heartburn, regurgitation, bloating, and swallowing difficulties as per the following scale: 0 - no symptoms; 1 - noticeable, but not bothersome; 2 - noticeable, bothersome, but not every day; 3 - bothersome daily; 4 - bothersome and affects daily activities; 5 - incapacitating to do daily activities. The highest possible score for each symptom category was 30, while the lowest was 0. The maximum possible total score was 75 (indicating the worst symptoms), and the minimum possible score was 0 (indicating no symptoms). Scores ≤12 indicated mild symptoms, while scores >12 indicated moderate to severe symptoms. These scores were used to classify the data based on disease severity.

Details of the effect of treatment and its tolerability based on patients' and physicians' records were also analyzed. 

Endpoints

Primary Endpoints

Improvement in GERD symptoms was measured by changes in symptom scores at the end of one week. The responder's rate, i.e., a reduction of 50% or more in the total symptom score from baseline to the end of one week, was also calculated based on the symptom scores [20,21].

Secondary Endpoints

Patient tolerability was assessed through the frequency and severity of adverse events and physician-reported outcomes.

Statistical analysis

Data measured on a continuous scale were expressed as mean ± standard deviation. Categorical data were represented as proportions or percentages. A paired t-test was carried out to compare the improvement in symptom scores at baseline and one week ± two days after sodium alginate antacid suspension. The statistical analysis was performed using SPSS 20. version (IBM, Inc., Armonk, US), and p-value <0.001 was considered to be significant.

Ethical considerations

This study was approved by the Independent Ethics Committee Dhanashree Hospital and registered with the Clinical Trial Registry of India (CTRI) with registration number CTRI/2024/07/070568.

## Results

Baseline demographics

Data from centers encompassing 10,000 patients who received sodium alginate antacid suspension for one week ± two days were retrieved and analyzed. Data for 7,192 patients for whom the records were available was further reviewed for the study. Based on the translation of symptoms into GERD symptom scores, data was categorized into two groups: patients with mild symptoms (total score ≤12) and those with moderate to severe symptoms (total score >12). Data from records for 6,246 patients with moderate-to-severe symptoms was further analyzed. The mean age of patients was 42.43 years. The other parameters are presented in Table 1.

**Table 1:** Measurements during general examination at baseline

Variable			n	Mean±SD
Gender	Male		3944	-
	Female		2199	
Pulse rate (beats/min)			3027	82.87±12.04
Body weight (kg)			3242	65.68±11.23
Height (cm)			3153	164.74±12.32
Blood pressure (mmHg)		Systolic	3102	127.50±13.74
		Diastolic	3090	83.59±13.96

Symptom relief at the end of treatment

The mean total heartburn score decreased significantly from baseline to the end of treatment, reflecting a significant reduction in heartburn symptoms. Similarly, there was a significant decrease in the mean total regurgitation score, indicating an improvement in regurgitation symptoms. The mean total swallowing and bloating scores also showed a significant reduction, demonstrating relief from these symptoms. Overall, the mean total score also reduced at the end of treatment, indicating an improvement across all measured parameters. Thus, treatment with sodium alginate antacid suspension led to substantial relief from GERD symptoms (Table *2*).

**Table 2:** Symptomatic relief with sodium alginate antacid suspension Paired t-test was performed and p-values were calculated. EOT - end of treatment

Parameter	Number of patients	Mean±SD		p-values	Mean reduction in symptom scores (%)
		Baseline	EOT (1 week ± 2 days)		
Total heartburn scores	6246	13.96±4.62	4.84±4.25	<0.001	65.32
Total regurgitation scores	6246	12.62±4.94	4.54±4.12	<0.001	64.02
Total swallowing and bloating scores	6246	5.84±3.08	2.12±2.26	<0.001	63.69
Total score	6246	32.42±10.86	11.51±9.82	<0.001	64.49

**Figure 1:**
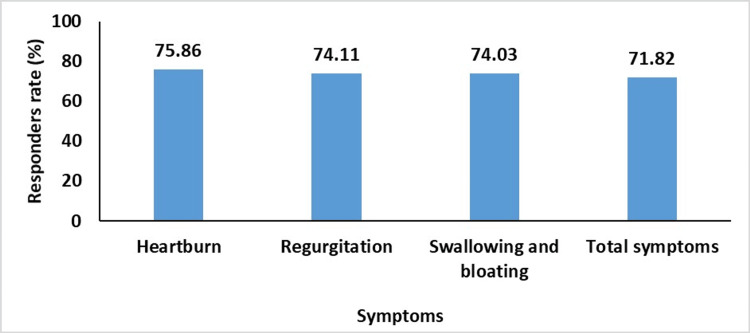
Responders rate for improvement in the symptoms of GERD GERD - gastroesophageal reflux disease

The responders' rate was >74% for all symptoms and 71.82% for the total symptom reduction within one week of treatment (Figure *1*). 

Patient-reported improvement in symptoms

**Figure 2:**
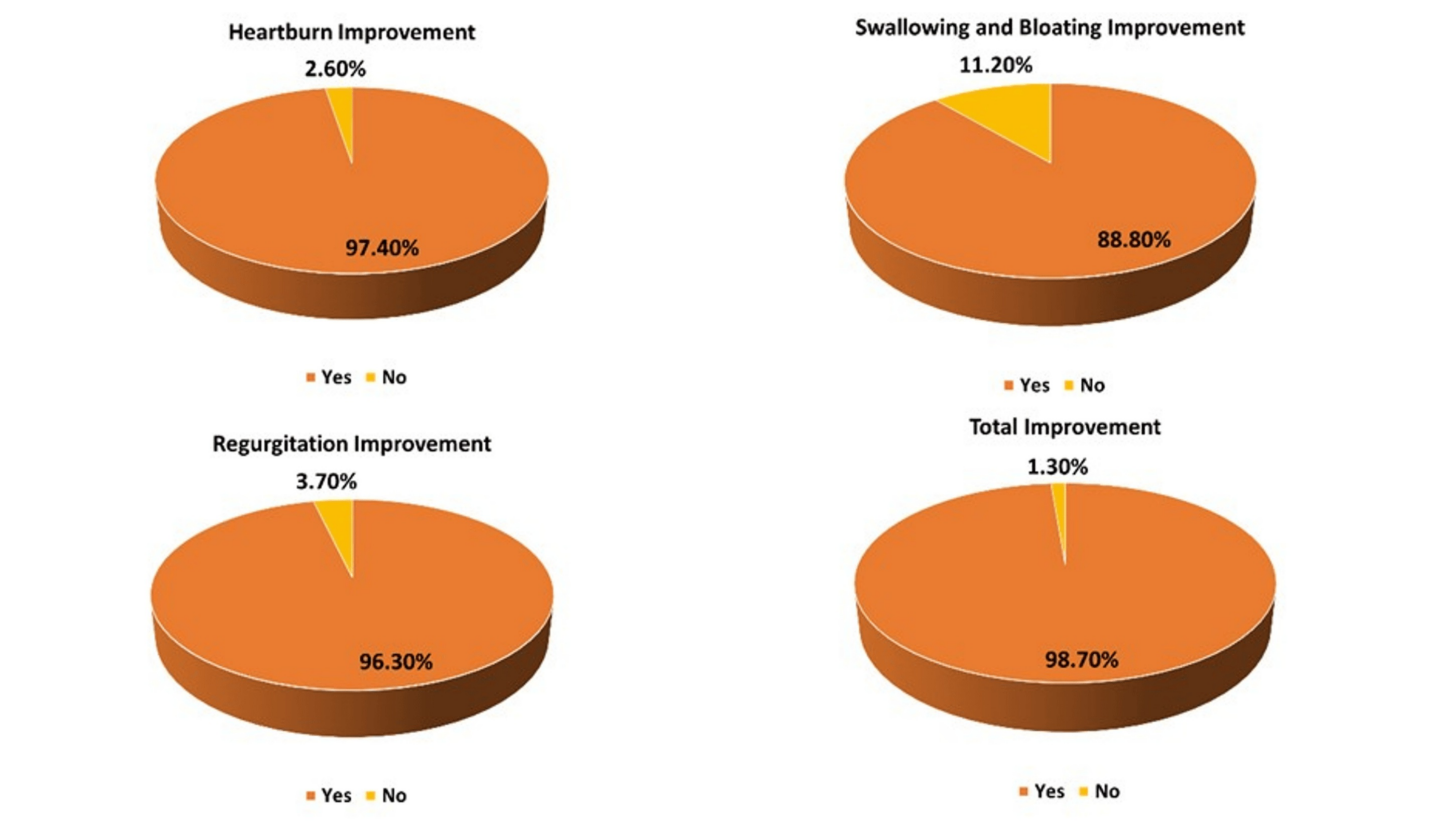
Patients reported clinically meaningful improvement in GERD symptoms GERD - gastroesophageal reflux disease

Patients reported clinically meaningful improvement in heartburn, regurgitation, and overall symptoms on treatment with sodium alginate antacid suspension (Figure *2*).

Tolerability of sodium alginate antacid suspension

Adverse effects were reported in 2.22%, 139 out of 6246 patients. Overall, no serious adverse reactions were reported.

Physician-reported outcomes

According to the physicians, sodium alginate antacid was effective in 96.64%, 4058 out of 4199 patients.

## Discussion

GERD is a prevalent condition and is typically characterized by symptoms such as heartburn and acid regurgitation [1]. These symptoms disrupt the daily lives of affected individuals by affecting physical activity, impairing social functioning, disturbing sleep, and decreasing productivity at work, thereby negatively impacting their quality of life [5,22-23]. Thus, rapid and effective alleviation of symptoms is important in GERD patients as it helps in improving quality of life [9].

Alginate antacids have an important place in GERD therapy due to their unique mechanism of action. These formulations form a viscous gel or raft that acts as a physical barrier, preventing gastric contents from refluxing into the esophagus [24-25]. This barrier is created swiftly, typically within seconds of administration, and can persist in the stomach for several hours [20]. This physical action provides rapid and prolonged symptom relief compared to traditional antacids. Alginate antacids have demonstrated effectiveness both as standalone treatments and as adjuncts to acid-suppressing medications, particularly for managing breakthrough symptoms along with PPIs [24].

The present study demonstrates that sodium alginate antacid suspension provides significant alleviation of GERD symptoms, such as heartburn, regurgitation, dysphagia, and bloating, within one week of initiating treatment. By the end of the treatment period, there were marked reductions in symptom scores. Over 90% of patients reported improvement in heartburn, regurgitation, and overall symptoms. Additionally, more than 75% of patients achieved at least a 50% reduction in heartburn, while over 70% experienced comparable reductions in regurgitation, dysphagia, and bloating. In terms of tolerability, the sodium alginate antacid suspension was generally well-tolerated, with only 2.22% of patients reporting adverse drug reactions.

The results of this study highlight the efficacy of sodium alginate antacid suspension in providing rapid and substantial relief from GERD symptoms in Indian patients. The significant symptom alleviation observed within one week, combined with a high rate of patient response and overall tolerability, suggests that alginate antacids are important for patients seeking prompt relief from their symptoms.

Large sample size and real-world evidence mimicking clinical settings are the strengths of this study. However, this study also has some limitations. Retrospective open-label study, missing data, and lack of medications co-prescribed are some important limitations of the study, which may affect the generalizability of the study results. Despite these multiple limitations, we believe that the study highlights some clinically relevant information for practicing clinicians.

## Conclusions

The present Indian real-world evidence highlights that sodium alginate antacid suspension offers rapid and significant relief from GERD symptoms, such as heartburn, regurgitation, dysphagia, and bloating, with noticeable improvements within one week of treatment.
